# Community-based benchmarking improves spike rate inference from two-photon calcium imaging data

**DOI:** 10.1371/journal.pcbi.1006157

**Published:** 2018-05-21

**Authors:** Philipp Berens, Jeremy Freeman, Thomas Deneux, Nikolay Chenkov, Thomas McColgan, Artur Speiser, Jakob H. Macke, Srinivas C. Turaga, Patrick Mineault, Peter Rupprecht, Stephan Gerhard, Rainer W. Friedrich, Johannes Friedrich, Liam Paninski, Marius Pachitariu, Kenneth D. Harris, Ben Bolte, Timothy A. Machado, Dario Ringach, Jasmine Stone, Luke E. Rogerson, Nicolas J. Sofroniew, Jacob Reimer, Emmanouil Froudarakis, Thomas Euler, Miroslav Román Rosón, Lucas Theis, Andreas S. Tolias, Matthias Bethge

**Affiliations:** 1 Institute for Ophthalmic Research, University of Tübingen, Tübingen, Germany; 2 Center for Integrative Neuroscience, University of Tübingen, Tübingen, Germany; 3 Bernstein Center for Computational Neuroscience, University of Tübingen, Tübingen, Germany; 4 Chan Zuckerberg Initiative, San Francisco, California, United States of America; 5 Janelia Research Campus, Howard Hughes Medical Institute, Ashburn, Virginia, United States of America; 6 Unit of Neuroscience Information and Complexity, Centre National de la Recherche Scientifique, Gif-sur-Yvette, France; 7 Bernstein Center for Computational Neuroscience and Institute for Theoretical Biology, Humboldt-Universität zu Berlin, Berlin, Germany; 8 Research Center Caesar, an associate of the Max Planck Society, Bonn, Germany; 9 Department of Electrical and Computer Engineering, Technical University of Munich, Munich, Germany; 10 Independent Researcher, San Francisco, California, United States of America; 11 Friedrich Miescher Institute of Biomedical Research, Basel, Switzerland; 12 University of Basel, Basel, Switzerland; 13 Departments of Statistics and Neuroscience, Grossman Center for the Statistics of Mind, and Center for Theoretical Neuroscience, Columbia University, New York, New York, United States of America; 14 Institute of Neurology, University College, London, United Kingdom; 15 Departments of Mathematics and Computer Science, Emory University, Atlanta, United States of America; 16 Neurobiology and Psychology, Jules Stein Eye Institute, Biomedical Engineering Program, David Geffen School of Medicine, University of California, Los Angeles, California, United States of America; 17 Departement of Computer Science, Yale University, New Haven, Connecticut, United States of America; 18 Department of Neuroscience, Baylor College of Medicine, Houston, Texas, United States of America; 19 Center for Neuroscience and Artificial Intelligence, Baylor College of Medicine, Houston, Texas, United States of America; 20 Division of Neurobiology, Department Biology II, LMU Munich, Munich, Germany; 21 Twitter, London, United Kingdom; 22 Department of Electrical and Computer Engineering, Rice University, Houston, Texas, United States of America; 23 Institute of Theoretical Physics, University of Tübingen, Tübingen, Germany; University College London, UNITED KINGDOM

## Abstract

In recent years, two-photon calcium imaging has become a standard tool to probe the function of neural circuits and to study computations in neuronal populations. However, the acquired signal is only an indirect measurement of neural activity due to the comparatively slow dynamics of fluorescent calcium indicators. Different algorithms for estimating spike rates from noisy calcium measurements have been proposed in the past, but it is an open question how far performance can be improved. Here, we report the results of the *spikefinder* challenge, launched to catalyze the development of new spike rate inference algorithms through crowd-sourcing. We present ten of the submitted algorithms which show improved performance compared to previously evaluated methods. Interestingly, the top-performing algorithms are based on a wide range of principles from deep neural networks to generative models, yet provide highly correlated estimates of the neural activity. The competition shows that benchmark challenges can drive algorithmic developments in neuroscience.

## Author summary

Two-photon calcium imaging is one of the major tools to study the activity of large populations of neurons in the brain. In this technique, a fluorescent calcium indicator changes its brightness when a neuron fires an action potential due to an associated increase in intracellular calcium. However, while a number of algorithms have been proposed for estimating spike rates from the measured signal, the problem is far from solved. We organized a public competition using a data set for which ground truth data was available. Participants were given a training set to develop new algorithms, and the performance of the algorithms was evaluated on a hidden test set. Here we report on the results of this competition and discuss the progress made towards better algorithms to infer spiking activity from imaging data.

## Introduction

Two-photon calcium imaging has become a standard tool to probe the function of neural circuits and to study computations in neuronal populations [1, 2]. Indeed, the latest advances in scanning technologies make it now possible to record neural activity from hundreds or even thousands of cells simultaneously [3–5]. However, the resulting fluorescence signal is only an indirect measurement of the underlying spiking activity, as it reflects the comparatively slow cellular dynamics of cellular calcium and the fluorescent calcium indicators [6–8]. Thus, to relate large-scale population recordings to the spiking activity of neural circuits we fundamentally require techniques to infer spike rates from the fluorescent traces.

Over the past decade, a number of algorithms for solving this problem have been proposed. Many of them assume a forward generative model of the calcium signal and attempt to invert it to infer spike rates. Examples of this approach include deconvolution techniques [9, 10], template-matching [4, 11] and approximate Bayesian inference [6, 12, 13]. Such forward models incorporate a priori assumptions about how the measured signal is generated, e.g. about the shape of the calcium fluorescence signal induced by a single spike and the statistics of the noise. In contrast, comparatively few groups have attempted to solve the problem through supervised learning [14, 15], where a machine learning algorithm is trained to infer the spike rate from calcium signal using simultaneously recorded spike and calcium data for training.

Despite this progress, it is still an open question whether current algorithms already achieve the best possible performance for the task, or whether the observed performance can still be improved upon by algorithmic developments. To answer this question, we organized the *spikefinder* challenge. This challenge aimed at two goals: (1) provide a standardized framework to evaluate existing spike inference algorithms on identical data and (2) catalyze the development of new spike inference algorithms through crowd-sourcing. Such challenges have been used successfully in machine learning, computer vision or physics to drive algorithmic developments [16, 17]. We present ten of the submitted algorithms which show improved performance compared to previously evaluated methods [15]. Interestingly, the top-performing algorithms are based on a range of principles from deep neural networks to generative models, yet provide highly correlated estimates of the neural activity.

## Results

For the *spikefinder* challenge, we used five benchmark data sets consisting in total of 92 recordings from 73 neurons, acquired in the primary visual cortex and the retina of mice (see Table 1). In brief, data sets I, II and IV were collected with OGB-1 as a calcium dye, while data sets III and V were collected with the genetically encoded indicator GCamp6s. Similarly, there were differences in scanning method and scan rate between the data sets: For example, data set I was recorded using 3D AOD scanners at very high scan rates [3], while data set II was recorded using conventional galvo-scanners at fairly low speed. For all data sets, calcium imaging had been performed simultaneously with electrophysiological recordings allowing to evaluate the performance of spike rate inference algorithms on ground truth data [15]. Importantly, all data was acquired at a zoom factor typically used during population imaging experiments, ensuring that all benchmark results reflect performance under the typical use-case conditions.

**Table 1:** Overview over datasets with training and test data used in the competition.

Dataset	Scan method	Indicator	Avg. scan rate (Hz)	N in training set	N in test set
I	3D AOD	OGB-1	322.5	11	5
II	galvo	OGB-1	11.8	21	10
III	resonant	GCamp6s	59.1	13	6
IV	galvo	OGB-1	7.8	6	3
V	resonant	GCamp6s	59.1	9	8

For the challenge, we split the data into a training and a test set, making sure that all recordings from a single neuron were either assigned to the training or the test set. For the training data, we made both the calcium and the spike traces publicly available, but kept the spike traces secret for the test data. Additionally, the publicly available data sets provided by the GENIE project [18] were available as training data. This allowed participants to adjust their models on the training data set, while avoiding overfitting to the specific benchmark data set providing a realistic estimate of the generalization performance. Participants could upload predictions for the spike rate generated by their algorithm on a dedicated website (see Methods) and see their performance on the training set during the competition phase. Results on the test set were not accessible to the participants during the competition. The primary evaluation measure for the competition was the Pearson correlation coefficient between the true spike trace and the prediction sampled at 25 Hz (equivalent to 40 ms time bins) as previously described [15].

We obtained 37 submissions, from which we selected all algorithms performing better than the spike-triggered-mixture model algorithm (STM), which had previously been shown to outperform other published algorithms on this data [15]. In addition, if there were multiple submissions from the same group, we used the one with the highest correlation on the test set. This resulted in a total of ten algorithms that we studied in greater detail and that are included in this paper. Notebooks and code showing how to run the individual algorithms are available at https://github.com/berenslab/spikefinder_analysis (see Table 2). While seven of these algorithms were designed specifically for the purpose of the challenge, three were heavily based on methods published previously (see Table 2 for overview).

**Table 2:** Overview over submitted algorithms and key properties.

Team	Contributors	new?	Language	Type
1	T. Deneux	-[12]	Matlab	generative
2	N. Chenkov, T. McColgan	+	Python	supervised
3	A. Speiser, J. Macke, S. Turaga	+	Python	supervised
4	P. Mineault	+	Python	supervised
5	P. Rupprecht, S. Gerhard, R. W. Friedrich	+	Python	supervised
6	J. Friedrich, L. Paninski	-[13]	Python	generative
7	M. Pachitariu	-[28]	Matlab	supervised
8	B. Bolte	+	Python	supervised
9	T. Machado, L. Paninski	+	Python	generative
10	D. Ringach	+	Matlab	supervised

Interestingly, these submissions include algorithms based on very different principles: some of the algorithms built on the classical generative models of spike-induced calcium dynamics [6], while others relied on purely data-driven training of deep neural networks or pursued hybrid strategies. Algorithms based on generative models of the calcium fluorescence include the MLspike algorithm by Team 1 [12], which performs efficient Bayesian inference in a biophysical model of measured fluorescence including a drifting baseline and nonlinear calcium to fluorescence conversion (for a detailed description of each algorithm, see S1 Text). Within the same group of algorithms, Team 6 took a decidedly different approach, approximating the calcium fluorescence by an autoregressive process and finding the spike trains by solving a non-negative sparse optimization problem [13, 19]. A similar approach was taken by Team 7, who used *L*_0_-deconvolution in a linear model of calcium fluorescence with exponential calcium filters.

In contrast, many other algorithms took a purely data-driven approach [15] and trained different variants of deep neural networks to learn the relationship between measured spike and calcium traces. For example, the algorithm by Team 2 used a straightforward network architecture with eight convolutional layers with consecutively smaller convolutional filters and one intermediate recurrent LSTM layer. The filters learned in the first layer provide a rich basis set for different spike-calcium relationships (see S1 Text). Similarly, the algorithm by Team 5 used fairly standard components, consisting of convolutional and max-pooling layers. In contrast, the algorithms proposed by Teams 3, 4, and 8 combined more involved elements such as residual blocks [20] or inception cells [21]. The key features of the different DNN-based approaches are summarized in Table 3.

**Table 3:** Overview over different strategies used by DNN-based algorithms. Architecture briefly summarizes main components. conv: convolutional layers, typically with non-linearity; lstm: recurrent long-short-term memory unit; residual: residual blocks; max: max-pooling layers; inception: inception cells. For details, refer to the descriptions of the algorithms in the supplementary material.

Team	Architecture	Optimizer	Dropout	Cost	dataset specific
2	conv / lstm	Adam	yes	correlation	indicator
3	RNN/CNN	Adam		cross-entropy	separate
4	residual / lstm	Adam	yes	scaled SSE	transfer
5	conv / max	Adagrad	no	MSE	embedding
8	inception	Adam	yes	correlation	embedding

**Fig 1:**
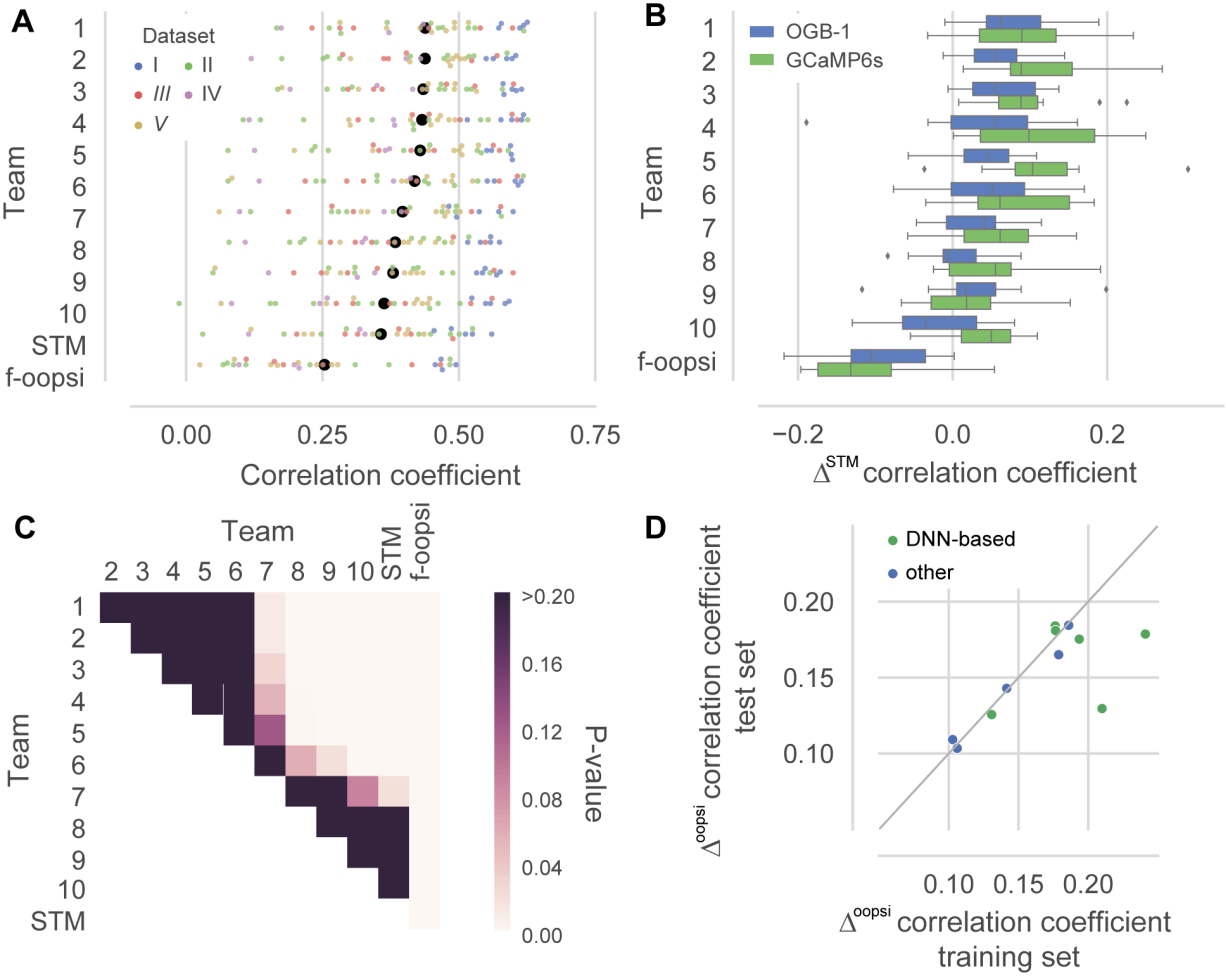
Contributed algorithms outperform state-of-the-art. **A.** Correlation coefficient of the spike rate predicted by the submitted algorithms (evaluated at 25 Hz, 40 ms bins) on the test set. Colors indicate different data sets (for details, see Table 1). Data sets I, II, and IV were recorded with OGB-1 as indicator, III and V with GCaMP6s. Black dots are mean correlation coefficients across all *N* = 32 cells in the test set. Colored dots are jittered for better visibility. STM: Spike-triggered mixture model [15]; f-oopsi: fast non-negative deconvolution [9] **B.** Difference in correlation coefficient on the test set to the STM, split by the calcium indicator used in the data set. **C.** P-values for difference in mean correlation coefficient on the test set for all pairs of algorithms (Repeated measured ANOVA, *N* = 32 cells, main effect of algorithm: P < 0.001, shown are p-values for post-hoc pairwise comparisons, corrected using Holm-Bonferroni correction) **D.** Difference in correlation coefficient split by algorithm type on the training and test set, respectively, to the f-oopsi-result correcting for systematic differences between the training and the test set.

The best algorithm increased the average correlation on the test set from 0.36 by 0.08 to 0.44 compared to the STM (Fig 1A and 1B; Table 4). This corresponds to an increase of more than 40% in variance explained for the best algorithms, similar to the improvement seen between the STM algorithm and f-oopsi (see Table 4 and ref. [15]). For all algorithms, performance varied substantially between data sets with the best results observed on data set I. Interestingly, performance gains were typically larger on GCaMP6 than on OGB-1 data sets (Fig 1B). Surprisingly, the top group of six algorithms performed equally well, despite using very different methodologies. Indeed, when we computed a repeated measures ANOVA, we were not able to distinguish the first six algorithms during post-hoc testing (Fig 1C). In addition, we evaluated to what extent the algorithms overfitted the training data. For example, it is possible that algorithms extracted peculiarities of the training data that did not transfer to the test data, resulting in artificially high correlation coefficients on the training data. We found that most algorithms showed similar performance for both the training and the test set, with evidence for overfitting in some of the DNN-based algorithms (Fig 1D).

**Table 4:** Summary of algorithm performance. Δ correlation is computed as the mean difference in correlation coefficient compared to the STM algorithm. Δ var. exp. in % is computed as the mean relative improvement variance explained (*r*^2^). Note that since variance explained is a nonlinear function of correlation, algorithms can be ranked differently according to the two measures. All means are taken over *N* = 32 recordings in the test set, except for training correlation, which is computed over *N* = 60 recordings in the training set.

Team	train correlation	test correlation	Δ correlation	Δ var. exp. %	AUC	Info
1	0.4823	0.4382	0.0810	44.1	0.846	2.922
2	0.4727	0.4378	0.0806	42.0	0.846	3.118
3	0.4730	0.4347	0.0775	41.8	0.851	3.085
4	0.5374	0.4325	0.0753	42.9	0.815	2.816
5	0.4900	0.4291	0.0719	40.5	0.842	2.725
6	0.4752	0.4188	0.0617	36.5	0.822	2.778
7	0.4379	0.3967	0.0395	22.2	0.829	2.797
8	0.5063	0.3833	0.0261	13.1	0.816	2.415
9	0.4271	0.3794	0.0222	13.5	0.815	2.816
10	0.3992	0.3629	0.0058	11.0	0.784	2.253
STM	0.4024	0.3572			0.821	2.468
f-oopsi	0.2964	0.2538	-0.1010	-40.4	0.658	1.107

**Fig 2:**
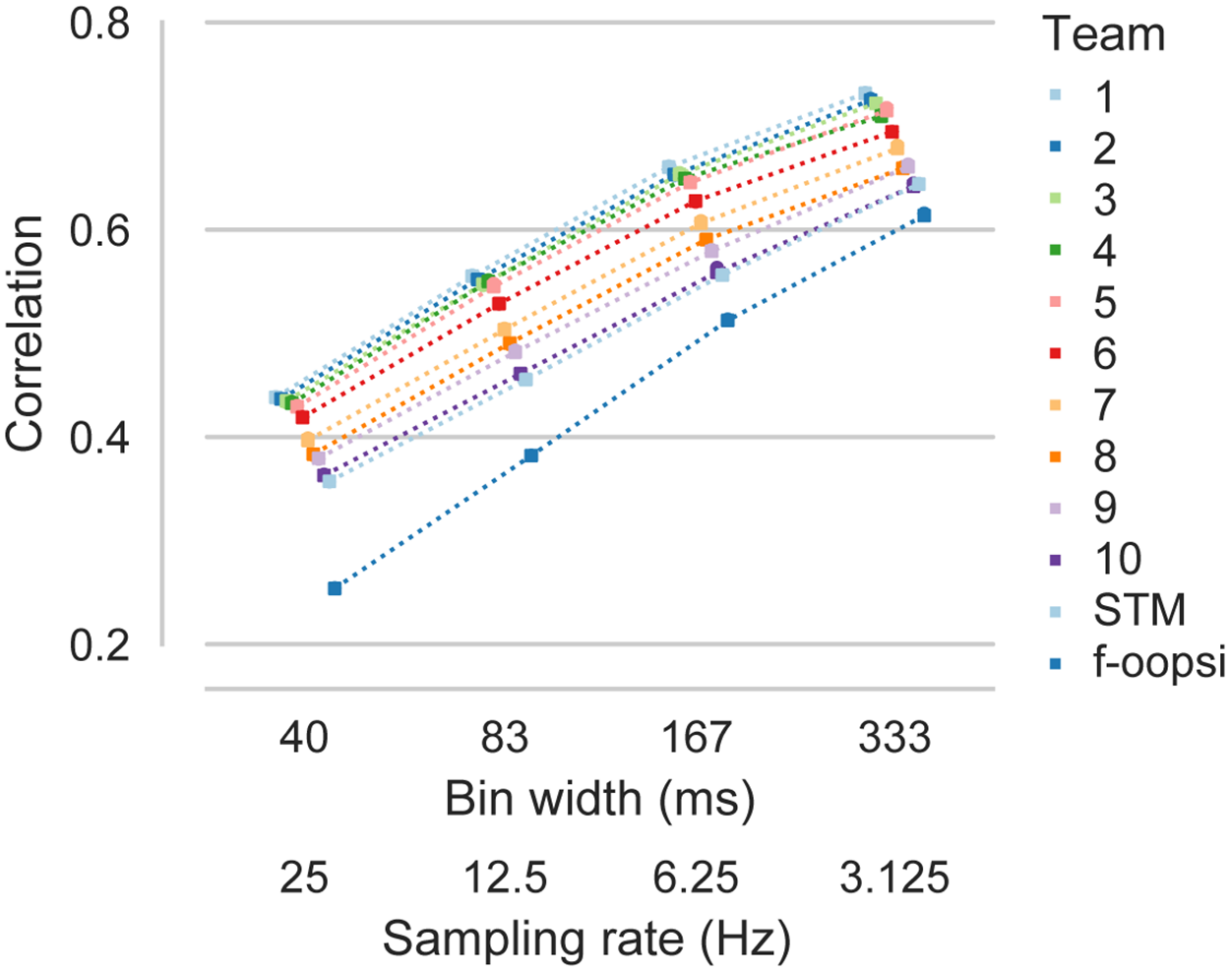
Temporal resolution does not change the ranking of algorithms. Mean correlation between inferred and true spike rates evaluated at different temporal resolution/sampling rate on all *N* = 32 cells in the test set. Colors indicate different algorithms. Colored dots are offset and connected for better visibility. STM: Spike-triggered mixture model [15]; f-oopsi: fast non-negative deconvolution [9].

**Fig 3:**
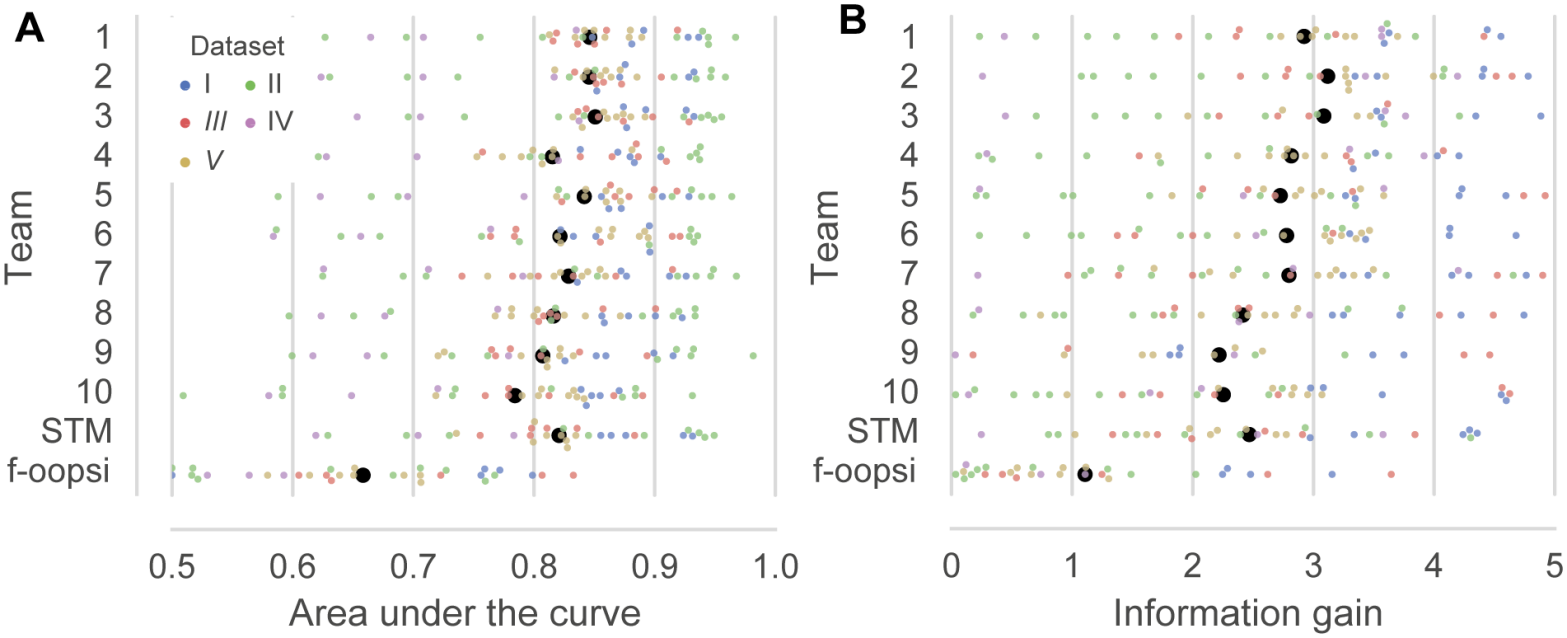
Different spike inference metrics reach similar conclusions. **A.** Area under the curve (AUC) of the inferred spike rate used as a binary predictor for the presence of spikes (evaluated at 25 Hz, 50 ms bins) on the test set. Colors indicate different datasets. Black dots are mean correlation coefficients across all *N* = 32 cells in the test set. Colored dots are jittered for better visibility. STM: Spike-triggered mixture model [15]; f-oopsi: fast non-negative deconvolution [9] **B.** Information gain of the inferred spike rate about the true spike rate on the test set (evaluated at 25 Hz, 40 ms bins).

To explore the generality of our findings, we additionally analyzed the performance of the algorithms at different temporal resolutions and using different evaluation measures. To this end, we computed the average correlation coefficient between the inferred and the true spike rates for bins of 40, 83, 167 and 333 ms, respectively (Fig 2). As expected, the average correlation increased with increasing bin width (e.g. for algorithm by team 1: 0.44 to 0.73). Interestingly, the rank of the algorithms was consistent across bin widths. In addition, we evaluated the performance of the algorithm using the AUC and information gain (Fig 3, Table 4, see Methods). The AUC measures the accuracy with which the presence of spiking in a given bin is detected, neglecting differences in the number of spikes. The information gain provides a model-based estimate of the amount of information about the spike rate extracted from the calcium trace [15]. The ranking of the algorithms was broadly consistent with the ranking based on correlation, despite minor differences.

**Fig 4:**
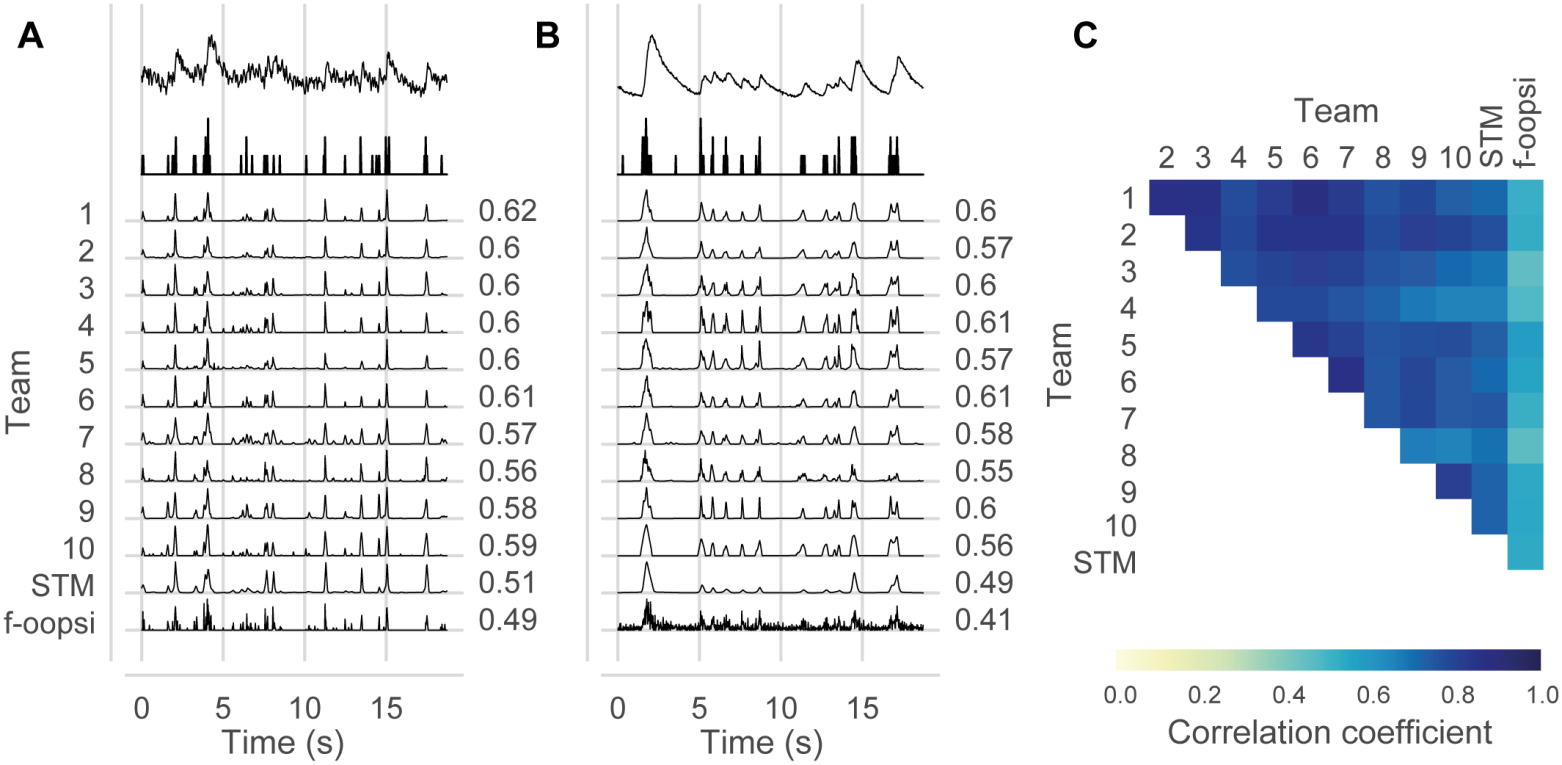
Top algorithms make highly correlated predictions. **A.-B.** Example cells from the test set for dataset 1 (OGB-1) and dataset 3 (GCaMP6s) show highly similar predictions between most algorithms. **C.** Average correlation coefficients between predictions of different algorithms across all cells in the test set at 25 Hz (40 ms bins).

As the algorithms in the top group used a range of algorithmic strategies, we wondered whether they also made different predictions, e.g., each capturing certain aspects of the spike-calcium relationship but not others. However, the predictions of the different algorithms were typically very similar with an average pairwise correlation coefficient among the first six algorithm of 0.82±.04 (mean ± SD, Fig 4). Also, averaging the top six predictions in an ensembling approach did not yield substantially better performance ($\bar{c}=0.4436$ compared to $\bar{c}=0.4382$ for Team 1). This indicates that despite their different algorithmic strategies, all algorithms captured similar aspects of the spike-fluorescence relationship.

## Discussion

In summary, the *spikefinder* challenge has shown that a community competition making use of suitable benchmark data can catalyze algorithmic developments in neuroscience. The challenge triggered a range of new and creative approaches towards solving the problem of spike rate inference from calcium data and improved the state-of-the-art substantially. The challenge did not distill the optimal strategy out of the different possible algorithmic approaches, something we had initially hoped for; rather, it showed that—given the current data—a range of approaches yield very similar outcomes.

### Different algorithmic strategies for spike rate inference

Interestingly, algorithms based on very different approaches yielded very similar performance. For example, algorithms based on generative models such as those by Team 1 and 6 perform on par with—in principle—more flexible deep learning-based approaches. Each algorithm comes with their own advantages and disadvantages regarding speed, interpretability, and incorporation of prior knowledge. For example, training the DNN-based models can be computationally quite costly and their efficient use may require specialized hardware such as GPUs. In practice, when a trained algorithm is applied to infer spike rates, we found all DNN-based method comparably efficient with a run time of less than a second per recording. With supervised methods, care has to be taken when using complex models to avoid overfitting the training set, as this could lead to false confidence about the prediction performance on new data. In fact, we observed quite heavy overfitting for two of the DNN-based approaches (Fig 1D). Nevertheless, supervised spike inference algorithms have been shown to generalize well to new data sets for which no data had been used during training [15], indicating that adapting supervised algorithms to new settings like indicators with different dynamics should be reasonably straightforward. In contrast, the algorithms based on generative models may be less easily adapted to novel settings as indicator dynamics, saturation or adaption effects and noise properties need to first be accurately assessed—simply swapping the measured calcium transient from isolated spikes may not be sufficient. In addition, inference in such models can be more time consuming as shown by the performance of the MLspike algorithm with an average of 15 seconds per recording. Hybrid approaches such as pursued here by Team 9 or more recently by [22] may offer a way towards combining the respective strengths of both approaches.

### Is spike rate inferences saturated?

The *spikefinder* challenge raises the question of what the actual performance bound of an ideal decoder is. Model simulations can help to answer these questions [8, 12], but their interpretation is limited by the accuracy of the model regarding indicator dynamics, noise structure, and other experimental factors [15]. For example, in vitro recordings zooming in on individual neurons will have a different maximal performance than recordings in awake, behaving animals. Of course, the achievable upper bound on performance always depends on the desired temporal resolution (Fig 2) and experimental factors. For example, cells in data set I recorded at very high sampling rates using 3D AOD scanning yielded on average much higher correlation than neurons recorded using the same indicator in the same area with much lower scan rate (Fig 1A). It remains to be seen whether new and larger data sets of simultaneously recorded and imaged neurons will yield further improvements and distinguish more clearly between different algorithmic strategies. It will also be interesting to see whether new indicators will allow for more precise spike rate inference.

### Evaluation of spike rate inference

We also considered the AUC and information gain as alternatives to our primary evaluation measure, the correlation coefficient. While the latter is easy to interpret and more sensitive than the AUC, it is still invariant under global scaling of the predicted spike rate [15]. Although information gain as a model based measured is considered a canonical model comparison criterion for probabilistic predictions [15, 23], it can be more difficult to interpret than correlation coefficients or AUC.

In general, all three measures yielded similar estimates of the ranking of the algorithms, with the AUC resolving the present differences least. In fact, different metrics can in principle lead to different conclusions about which algorithm is optimal since the metric contains part of the task specification [24]. Metrics for spike rate inference are a matter of current debate in the literature—see for example refs. [5, 25] for recent proposals.

### Design considerations for future challenges

In addition to improving on the state-of-the-art, competitions such as the *spikefinder* challenge can boost standardization of algorithms, something that has been lacking from neuroscience analysis [26]. For example, several of the processing choices made for this challenge triggered a debate among the submitting teams as to their utility and practicality. For example, we resampled all data to 100 Hz for ease of comparison, which induced problems for some of the submitted algorithms through the properties of the used filter. In addition, most participating teams found it necessary to introduce means of adapting the model parameters to the specific data set. These differences may have been introduced through different preprocessing procedures in the labs that contributed data and even between different scanning methods and speeds within the same lab (3D AOD vs. galvo scanning vs. resonant scanning). Even greater care should be taken to avoid such confounds in future competitions on this topic. In particular, a future challenge should explicitly address the potential of each algorithm to easily adapt to a data set not previously seen as part of the training set, testing for the transfer learning capabilities of each algorithm. It would also be interesting to explicitly evaluate algorithms for different recording conditions (e.g. in-vitro vs. awake), as the difference in recording conditions could even make different algorithmic strategies optimal.

Finally, the challenge was performed on traces extracted from the raw imaging data by averaging all the pixels within manually placed regions-of-interest (ROIs). It is thus possible that the extracted signals contain contamination from the neuropil or were suboptimally placed, a problem tackled by methods that combine ROI placement and calcium-trace extraction in a single algorithm [27, 28]. However, at least for data with simultaneous imaging and electrophysiological recordings, it is not clear how methods integrating ROI placement and spike rate extraction should be evaluated and compared to regular data, since the recording electrode is always present in the picture, adding a confound to automated ROI extraction through the different image statistics.

### Conclusion

We believe that quantitative benchmarks are an essential ingredient for progress in the field, providing a reference point for future developments and a common standard with regards to how new algorithms should be evaluated. We strongly believe that many fields of computational neuroscience can benefit from community-based challenges to assess where the field stands and how it should move forward. As for the problem of spike rate inference from two-photon imaging, the *spikefinder* challenge should not be considered the last word in this matter: More comprehensive data sets and new functional indicators may require organizing another round of community-based development, further pushing the boundaries of what is attainable. Which algorithm to choose? The answer to that depends on a lot of factors, including performance, desired programming language, envisioned run time and not the least the simplicity of the method—certainly, an algorithm consisting of ten simple lines of code like that by team 10 is more intuitive than a highly nonlinear DNN. The algorithms submitted as part of this challenge offer a range of options regarding these criteria and will provide a solid basis to further advance the field.

## Methods

### Data

The challenge was based on data sets collected for a previous benchmarking effort [15] and the publicly available cai-1 data set from crcns.org [18]. Details about the recording region, scan method, indicators, scan rate and cell numbers are summarized in Table 1 and described in detail in Theis et al. (2016). All data was resampled to 100 Hz independent of the original sampling rate. Upon request during the challenge, we made the data available at the native sampling rate.

### Challenge organization

For the challenge, we split the available data into training and test sets (see Table 1). The training set contained both calcium and spike data, while for the test set, only calcium data was available during the challenge period. We made sure that multiple recordings from individual neurons contained in some data sets were either assigned to the training or the test set. The GENIE datasets were only used as training data, since they are completely publicly available and consist of recordings from individual zoomed-in cells.

The data and instructions were available on a dedicated website, based on an open-source web framework (https://github.com/codeneuro/spikefinder). There was a discussion board linked from the website to allow for questions and discussion among participants. Each team could make multiple submissions, but during the challenge period, only results on the training set were shown. The challenge ran from 30/11/2016 to 04/05/2017.

### Algorithms

The submitted algorithms are described in detail in the Appendix. For comparison, we used publicly available implementations of the STM algorithm [15] and fast-oopsi [9]. STM parameters were optimized on the entire training set.

### Evaluation

The evaluation of the submissions was done in Python using Jupyter notebooks. All evaluation functions and notebooks are available at https://github.com/berenslab/spikefinder_analysis.

We used the correlation coefficient *c* between the inferred and the real traces resampled to 25 Hz (40 ms time bins) as primary quality measure. To make the observed increase in correlation more interpretable, we converted it to variance explained *r*^2^ and report the improvement in performance as the average increase in variance explained compared to the STM algorithm:

$$\begin{array}{c} 100\cdot (<\frac{c_{algo}^{2}}{c_{STM}^{2}}>-1)\% \end{array}$$

Here, <> denotes an average over cells, omitting the dependence of *c* on cells for clarity. For completeness, we also computed the area under the ROC curve (AUC) and the information gain as in ref. [15]. We used the roc_curve function from scikit-learn [29] to compute the AUC for classifying whether or not a spike was present in a given bin. Assuming Poisson statistics, independence of spike counts in different bins, an average firing rate λ and a predicted firing rate of λ_*t*_ at time *t*, the expected information gain (in bits per bin) can be estimated as

$$\begin{array}{c} I_{g}=\frac{1}{T}\sum_{t}k_{t}\log_{2}\frac{\lambda_{t}}{\lambda }+\lambda -\frac{1}{T}\sum_{t}\lambda_{t} \end{array}$$

Since the different algorithms were not necessarily optimized for this model, we transformed the predicted firing rate λ_*t*_ using a piecewise linear monotonically increasing function *f* optimized to maximize the information gain across all cells [15].

We used the R package afex to compute a repeated measures ANOVA on the correlation coefficients with within-subject factor algorithm and cells as subjects. Pairwise comparisons between algorithms were performed using the lsmeans package with Holm-Bonferroni correction for 66 tests.

## Supporting information

S1 Text Detailed description of all algorithms. The supplementary file contains detailed descriptions of all algorithms submitted as part of the spikefinder challenge.
(PDF)

## References

[1] Kerr JN, Denk W. Imaging in vivo: watching the brain in action. Nature Reviews Neuroscience. 2008;9(3):195–205. doi: 10.1038/nrn2338 18270513 10.1038/nrn2338

[2] Peron S, Chen TW, Svoboda K. Comprehensive imaging of cortical networks. Current Opinion in Neurobiology. 2015;32:115–123. doi: 10.1016/j.conb.2015.03.016 25880117 10.1016/j.conb.2015.03.016

[3] Cotton RJ, Froudarakis E, Storer P, Saggau P, Tolias AS. Three-dimensional mapping of microcircuit correlation structure. Frontiers in neural circuits. 2013;7:151 doi: 10.3389/fncir.2013.00151 24133414 10.3389/fncir.2013.00151 PMC3794294

[4] Grewe BF, Langer D, Kasper H, Kampa BM, Helmchen F. High-speed in vivo calcium imaging reveals neuronal network activity with near-millisecond precision. Nature methods. 2010;7(5):399 doi: 10.1038/nmeth.1453 20400966 10.1038/nmeth.1453

[5] Pachitariu M, Stringer C, Harris KD. Robustness of spike deconvolution for calcium imaging of neural spiking. bioRxiv. 2017; 156786. doi: 10.1101/156786 10.1523/JNEUROSCI.3339-17.2018 PMC6136155 30082416

[6] Vogelstein JT, Watson BO, Packer AM, Yuste R, Jedynak B, Paninski L. Spike inference from calcium imaging using sequential Monte Carlo methods. Biophysical journal. 2009;97(2):636–55. doi: 10.1016/j.bpj.2008.08.005 19619479 10.1016/j.bpj.2008.08.005 PMC2711341

[7] Chen TW, Wardill TJ, Sun Y, Pulver SR, Renninger SL, Baohan A, et al Ultrasensitive fluorescent proteins for imaging neuronal activity. Nature. 2013;499(7458):295–300. doi: 10.1038/nature12354 23868258 10.1038/nature12354 PMC3777791

[8] Wilt B, Fitzgerald JE, Schnitzer MJ. Photon shot noise limits on optical detection of neuronal spikes and estimation of spike timing. Biophysical journal. 2013;104(1):51–62. doi: 10.1016/j.bpj.2012.07.058 23332058 10.1016/j.bpj.2012.07.058 PMC3540268

[9] Vogelstein JT, Packer AM, Machado Ta, Sippy T, Babadi B, Yuste R, et al Fast nonnegative deconvolution for spike train inference from population calcium imaging. Journal of neurophysiology. 2010;104(6):3691–704. doi: 10.1152/jn.01073.2009 20554834 10.1152/jn.01073.2009 PMC3007657

[10] Yaksi E, Friedrich RW. Reconstruction of firing rate changes across neuronal populations by temporally deconvolved Ca 2+ imaging. Nature methods. 2006;3(5):377 doi: 10.1038/nmeth874 16628208 10.1038/nmeth874

[11] Greenberg DS, Houweling AR, Kerr JN. Population imaging of ongoing neuronal activity in the visual cortex of awake rats. Nature neuroscience. 2008;11(7):749 doi: 10.1038/nn.2140 18552841 10.1038/nn.2140

[12] Deneux T, Kaszas A, Szalay G, Katona G, Lakner T, Grinvald A, et al Accurate spike estimation from noisy calcium signals for ultrafast three-dimensional imaging of large neuronal populations in vivo. Nature Communications. 2016;7:12190 doi: 10.1038/ncomms12190 27432255 10.1038/ncomms12190 PMC4960309

[13] Friedrich J, Zhou P, Paninski L. Fast online deconvolution of calcium imaging data. PLoS Comput Biol. 2017;13(3):e1005423 doi: 10.1371/journal.pcbi.1005423 28291787 10.1371/journal.pcbi.1005423 PMC5370160

[14] Sasaki T, Takahashi N, Matsuki N, Ikegaya Y. Fast and accurate detection of action potentials from somatic calcium fluctuations. Journal of neurophysiology. 2008;100(3):1668–1676. doi: 10.1152/jn.00084.2008 18596182 10.1152/jn.00084.2008

[15] Theis L, Berens P, Froudarakis E, Reimer J, Román Rosón M, Baden T, et al Benchmarking Spike Rate Inference in Population Calcium Imaging. Neuron. 2016;90(3):471–482. doi: 10.1016/j.neuron.2016.04.014 27151639 10.1016/j.neuron.2016.04.014 PMC4888799

[16] Russakovsky O, Deng J, Su H, Krause J, Satheesh S, Ma S, et al Imagenet large scale visual recognition challenge. International Journal of Computer Vision. 2015;115(3):211–252. doi: 10.1007/s11263-015-0816-y

[17] Adam-Bourdarios C, Cowan G, Germain C, Guyon I, Kégl B, Rousseau D. The Higgs boson machine learning challenge. In: NIPS 2014 Workshop on High-energy Physics and Machine Learning; 2015. p. 19–55.

[18] Svoboda K, Project G. Simultaneous imaging and loose-seal cell-attached electrical recordings from neurons expressing a variety of genetically encoded calcium indicators; 2015. Available from: http://dx.doi.org/10.6080/K02R3PMN.

[19] Friedrich J, Paninski L. Fast active set methods for online spike inference from calcium imaging. In: Advances In Neural Information Processing Systems; 2016. p. 1984–1992.

[20] He K, Zhang X, Ren S, Sun J. Deep residual learning for image recognition. In: Proceedings of the IEEE conference on computer vision and pattern recognition; 2016. p. 770–778.

[21] Szegedy C, Liu W, Jia Y, Sermanet P, Reed S, Anguelov D, et al. Going deeper with convolutions. CVPR; 2015.

[22] Speiser A, Yan J, Archer E, Buesing L, Turaga SC, Macke JH. Fast amortized inference of neural activity from calcium imaging data with variational autoencoders. In: Advances in Neural Information Processing Systems. vol. 30; 2017.

[23] Kuemmerer M, Wallis T, Bethge M. Information-theoretic model comparison unifies saliency metrics. Proceedings of the National Academy of Science. 2015;112(52):16054–16059. doi: 10.1073/pnas.1510393112 10.1073/pnas.1510393112 PMC4702965 26655340

[24] Kümmerer M, Wallis TS, Bethge M. Saliency Benchmarking: Separating Models, Maps and Metrics. arXiv preprint arXiv:1704.08615. 2017.

[25] Reynolds S, Schultz SR, Dragotti PL. CosMIC: A Consistent Metric for Spike Inference from Calcium Imaging. bioRxiv. 2017; 238592. doi: 10.1101/238592 10.1162/neco_a_01114 30021084

[26] Freeman J. Open source tools for large-scale neuroscience. Current Opinion in Neurobiology. 2015;32:156–163. doi: 10.1016/j.conb.2015.04.002 25982977 10.1016/j.conb.2015.04.002

[27] Pnevmatikakis EA, Soudry D, Gao Y, Machado TA, Merel J, Pfau D, et al Simultaneous Denoising, Deconvolution, and Demixing of Calcium Imaging Data. Neuron. 2016;89(2):285–299. doi: 10.1016/j.neuron.2015.11.037 26774160 10.1016/j.neuron.2015.11.037 PMC4881387

[28] Pachitariu M, Stringer C, Schröder S, Dipoppa M, Rossi LF, Carandini M, et al Suite2p: beyond 10,000 neurons with standard two-photon microscopy. bioRxiv. 2016; doi: 10.1101/061507

[29] Pedregosa F, Varoquaux G, Gramfort A, Michel V, Thirion B, Grisel O, et al Scikit-learn: Machine Learning in Python. Journal of Machine Learning Research. 2011;12:2825–2830.

