# Supplementary material for: Community-based benchmarking improves spike rate inference from two-photon calcium imaging data
Source: PLoS Comput Biol. 2018 May 21;14(5):e1006157. doi: 10.1371/journal.pcbi.1006157 (PMC5997358; doi:10.1371/journal.pcbi.1006157)
Supplement: S1 Text — The supplementary file contains detailed descriptions of all algorithms submitted as part of the spikefinder challenge. (PDF) [file pcbi.1006157.s001.pdf]

Supplementary Text for Community-based benchmarking improves spike rate inference from two-photon calcium imaging data

Berens et al.

May 9, 2018

For all algorithms, we denote the spike train $\boldsymbol{s}$, the fluorescence trace $\boldsymbol{f}$ and the underlying calcium signal $\boldsymbol{c}$, where applicable. We observe a total of T time bins, and the measurement in time bin t is written s_t, f_t and c_t, respectively.

Team 1 — T. Deneux The MLspike algorithm [1] is a model-based Bayesian inference algorithm. Similarly to the method by Vogelstein et al. [2], the conversion of neuronal spiking activity to calcium fluorescence is modeled by a biophysical dynamical system, and a two-ways filtering scheme is applied to estimate the hidden dynamics of the intracellular calcium concentration given the noisy fluorescence recording. MLspike implements two major improvements over previous models: The first one is an extension of the biophysical model including a slowly drifting baseline, which allows disentangling a wide range of noises often observed in the real data from the spike-related signals. The second one is to represent probabilities as dense arrays rather than using Monte-Carlo approximations, namely making MLspike a histogram filter instead of a particle filter, which improves both speed (at least for a model's hidden state dimension not greater than 2) and accuracy.

For the spikefinder competition, MLspike was set to estimate a-posteriori probabilities $\mathbb{E}(s|c)$ rather than maximum-a-posteriori spike trains $\arg\max_s p(s|c)$. The biophysical model entailed a drifting baseline and nonlinear calcium to fluorescence conversion (i.e. saturation for OGB dataset; polynomial supralinearity for GCaMP6 dataset), and therefore had 6 or 7 parameters. One of these parameters (the a-priori spiking rate) was fixed while the 5 or 6 remaining ones were estimated independently for each training dataset so as to maximize the match between the estimated and observed spikes. This was preferred to using MLspike's autocalibration method on each individual neuron, because the data appeared too noisy for this autocalibration to perform accurately. The match between the true and inferred spike rate was defined as the correlation after resampling to 25 Hz between the true spike train, and the best post-processed version of the estimated spike train, were postprocessing consisted in applying an additional temporal smoothing and a time shift to account for apparent differences between data sets. Once these optimizations were performed, the same model and postprocessing parameters were applied to the test data sets. Interestingly, the hyperparameter optimization strategy pursued here was very similar to that chosen by Team 6.

Code is available at https://github.com/MLspike.

Team 2 — N. Chenkov, T. McColgan This algorithm is based on a convolutional neural network, which receives the calcium signal and an index vector as input, denoting the data set the inputs come from. The network consists of eight convolutional layers and one recurrent layer (LSTM) (see Fig. S1A). We optimized the parameters by maximizing the Pearson correlation coefficient with the ground-truth spiking data at 25 Hz using the 'Adam' optimizer with 50 epochs.

The first layer consists of 10 units. Each unit uses a kernel with a width of 3 seconds (300 time steps) that is correlated with the input calcium signal. The learned kernels catch a basic repertoire of spike-related calcium dynamics (see Fig. S1A). The output of this layer is passed through a hyperbolic tangent activation function ('tanh'). This layer is followed by multiple convolutional layers with smaller kernel widths (100 and 50 ms, or 10 and 5 time steps, respectively) and with rectified linear activation functions ('ReLU'). The data set indicator is concatenated with the

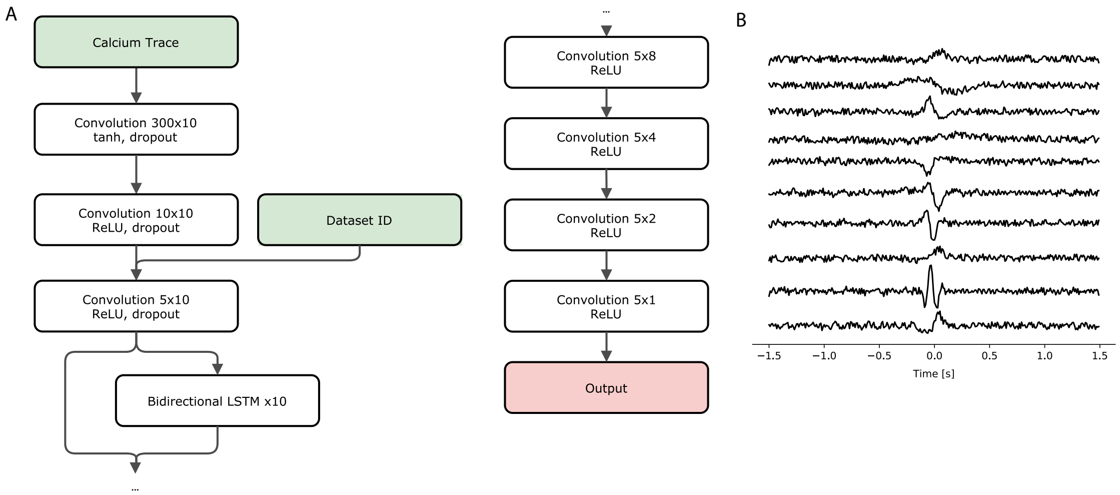

Figure 1: A. Network Architecture. In the convolutional layers the notation mxn denotes n units with kernel width of m time steps. B. Example of convolutional kernels learned by the model. The ten kernels of the first convolutional layer can describe a wide range of transient calcium dynamics.

output of the second layer and feed into the input of the third layer. We observed that the data set indicator improved the performance of the model, possibly setting different states of the recurrent layer dynamics. Moreover, a bidirectional LSTM layer is fed by the input of the third layer, and its output is added to the input to the fourth layer. The following four layers have decreasing size, with the last layer consisting of a single unit.

To distribute the amount of information that different units are carrying, dropout is applied at the output of the first five convolutional layers.

Code is available at `https://github.com/kleskjr/spikefinder-solution`.

Team 3 — A. Speiser, S. Turaga, J. H. Macke We trained neural networks consisting both of convolutional and recurrent layers to learn a mapping from fluorescence trace to neural spiking. In contrast to other methods, we trained the network to approximate a correlated posterior conditional probability distribution $q(s|f)$ of a spike-train $s_{t=0...T}$ given a fluorescence trace $f_{t=0...T}$. We use a recurrent layer to model an autoregressive conditional probability distribution to account for correlations in this posterior between spike probabilities and previously sampled spikes, similar to ref [3]. The temporal ordering over spikes is used to factorize the joint distribution as $q_\phi(s|f) = \prod_t q_\phi(s_t|f, s_0, ..., s_{t-1})$, by conditioning spike probabilities at t on all previously sampled spikes. The resulting stochastic RNN models a correlated posterior conditional distribution over spike trains (see Fig. S2). The stochastic RNN samples correlated spike trains (similar to MLSpike) which might be useful for certain applications. However, none of the performance measures used in this challenge are sensitive to this property, as they are based on marginal firing rate predictions.

As our objective function we used the binary cross entropy between our predictions and the true spike train to train the model in a supervised fashion

$$\min_\phi s_t \log\left(q_\phi(s_t|f, s_0, ..., s_{t-1})\right) + (1 - s_t)\log\left(1 - q_\phi(s_t|f, , s_0, ..., s_{t-1})\right). \tag{1}$$

In separate work[4], we developed an approach for training this network in an unsupervised fashion using variational auto-encoders [5] that can perform inference on a wide range of biophysical generative models (e.g. using the ones used by team 1). For the challenge, all available training data was labeled (i.e. ground truth spikes were provided), and we therefore trained the network using supervised learning.

Our architecture contains one forward running RNN that uses a multi-layer CNN with leaky ReLUs units to extract features from the input trace. The outputs of the forward RNN and CNN are transformed into Bernoulli spike probabilities through a dense sigmoid layer. Additional input is provided by a second RNN that runs backwards and also receives input from the CNN. Forward and backward RNN have a single layer with 128 gated recurrent units each[6]. In order to generate

a single marginal probability distribution $q_\phi(s_t|f)$ for evaluation, samples drawn by running the stochastic RNN 50 times were averaged. We trained one separate network for each dataset.

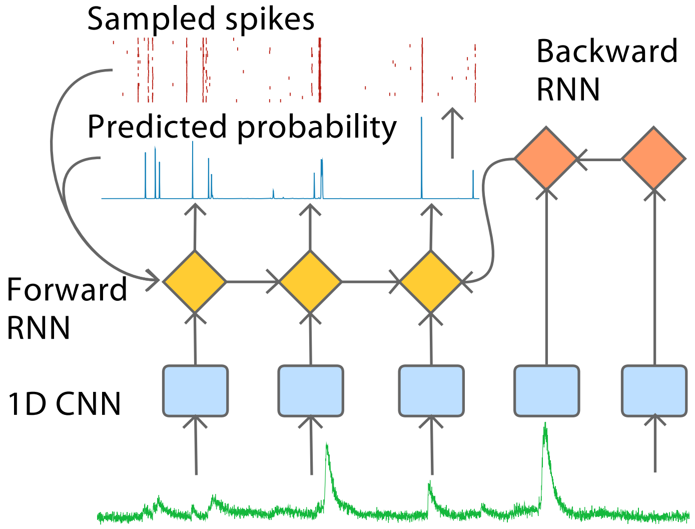

Figure 2: Network Architecture We use a multi-layer network architecture: Fluorescence-data is first filtered by a deep 1D convolutional network (CNN), providing input to a stochastic forward running recurrent neural network (RNN) which predicts spike-probabilities and takes previously sampled spikes as additional input. An additional deterministic RNN runs backward in time and provides further context.

To minimize the artifacts introduced by upsampling the data to a common imaging rate, we performed our own pre-processing (including percentile-detrending, normalizing and resampling) where we kept the fluorescence traces closer to the original recording frequency (i.e. 50, 50, 75, 12.5, 75 Hz for data sets 1-5 respectively). For the rare cases where the true spike train contains bins with multiple spikes at this rate, we clip the values to be binary. For training we split the traces into short snippets and arranged them into batches of size 10. For the rare cases where the true spike train contains bins with multiple spikes at this rate, we clip the values to be binary. Furthermore we used stochastic gradient descent with the Adam optimzer (using default parameters).

To find good hyperparameters we performed a small grid search on the following parameters and chose the best model using cross validation: learning rate $\{4e^{-4}, 1e^{-3}\}$, number of convolutional filters per layer $\{20/15/15/10, 35/30/20/10\}$, length of trace snippets $\{100, 200, 300\}$. Performance proved to be rather robust to the exact choice of hyperparameters.

The RNN produced suboptimal results on the fourth dataset (OGB, 7.8 Hz), and we therefore used a simple factorizing CNN on this data-set, at an upsampled rate of 4x the imaging rate. The RNN architecture achieved a correlation coefficient of 0.417 on the validation set against 0.455 when using the CNN.

Code is available at https://github.com/mackelab/DeepSpike.

Team 4 — P. Mineault This submission casts the problem as a supervised learning problem, where the goal is to estimate the parameters of a basis transformation g and an output non-linearity h such that a loss $L(\mu_t, s_t)$ between spike train s_t and prediction μ_t is minimized. g is given by a deep convolutional artificial neural network. The first layer of this network is a standard convolutional layer followed by a rectified linear (ReLU) nonlinearity[7], mapping each calcium time series f_t to 32 parallel time series indexed by k.

$$z^0_{tk} = (f_t * w^0_{jk} + \beta^0_k)^+$$

Here $(x)^+ \equiv \max(0, x)$ is the ReLU nonlinearity. We use a large window (33 time points, or 330 ms), batch normalization, as well as a dropout fraction of .3. This initial layer is followed by seven adjustment layers in the style of a residual network[8]:

$$z^{l+1}_{tk} = z^l_{tk} + \sum_m (z^l_{tm} * w^{l+1}_{tk} + \beta^{l+1}_k)^+$$

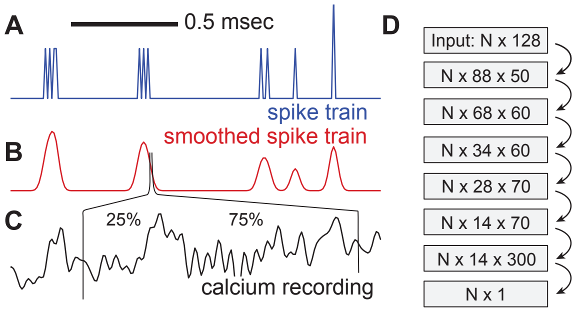

Layer	# filters/neurons	Filter size	# parameters
Convolutional	50	41	2.1k
Convolutional	60	21	63.1k
MaxPool	-	2	-
Convolutional	70	7	29.5k
MaxPool	-	2	-
Dense	300	1	21.3k
Dense	1	1	4.2k

Figure 3: The basic convolutional neural network. **A,B**. The spiking ground truth was smoothed to facilitate gradient descent. **C**. A 1.28 sec time window of the calcium trace was used to infer the spiking probability for each time point. **D**. The N inputs were transformed using a neural network with three convolutional layers. The numbers in the boxes indicate the output size of the respective layers.

These adjustment layers use smaller windows (9 time points, or 90 ms). The nonlinear component of each layer is batch normalized. Finally, the output is composed linearly via:

$$\eta_t = \sum_k z^7_{tk} w^7_k + \beta^7$$

The output nonlinearity h was given by a ReLU nonlinearity, such that $s_t = \eta_t^+$. We minimized a scaled sum of squared error criterion:

$$L = \sum_i \min_{\alpha_i} \frac{\sum_j (s^i_t - \alpha_i \mu^i_t)^2}{\sum_j (s^i_t)^2}$$

Here, i indexes different neurons and α_i is a set of scalars which are learned alongside the other parameters of the model (w and β). One can show that the loss is equivalent to $1 - \rho^2$, where ρ^2 is the square of the cosine similarity between prediction and spike train. The model was specified and fit using the `tf.contrib.learn` library in TensorFlow [9]. Model parameters were initialized with the Xavier method and fit using the Adam optimizer. One large model for all 10 recordings from the training set was fit (173 neurons) in this phase and goodness-of-fit was monitored on a leave-aside validation set to control overfitting by early stopping. Convergence took close to 200,000 iterations.

The model described so far uses fixed filters for each recording, and uses local information (≈ 1 second of data) to estimate spikes from calcium traces. To adapt filters, we learned long-range features with an unsupervised mixture density network [10, 11]. The model, a 3-layer recurrent neural network with 512 long-short-term memory (LSTM) nodes, was fit to the calcium data to obtain one 1536-dimensional latent vector per mini-batch, which was reduced to 32 dimensions by PCA after z-scoring. These features were processed by two fully connected layers to produce 4 hidden features γ_{tp}. These 4 hidden features were used to additively adapt the filters $w^0_{tjk} = \sum_p \text{softmax}(\gamma_{tp}) W_{jkp}$, in a manner similar to attention models.

Originally, one large model was fit for all recordings. We then created refined versions of this model for each of the 10 data sets by a transfer learning process. We took the large model with its learned parameters and ran up to 50,000 extra iterations of gradient descent on just the data from the kth dataset.

Code is available at
https://github.com/patrickmineault/spikefinder_submission

Team 5 — P. Rupprecht, S. Gerhard, R. W. Friedrich In order to infer a spike probability for time bin t (Fig. S3A), the calcium trace located around t was used, including 25% before and 75% after t, totaling to 128 samples, *i.e.*, 1.28 sec (Fig. S3C). A convolutional neural network was trained to use these 128-wide windows to predict the corresponding spiking probability. To facilitate gradient ascent, we smoothed the discrete spiking ground truth with a Gaussian filter ($\sigma = \sqrt{2}$ samples, Fig. S3B).

We implemented the convolutional neural network in Python using Keras [12] with the Tensorflow [9] backend (see Fig. 3 for the network architecture). The convolutional filter size, particularly

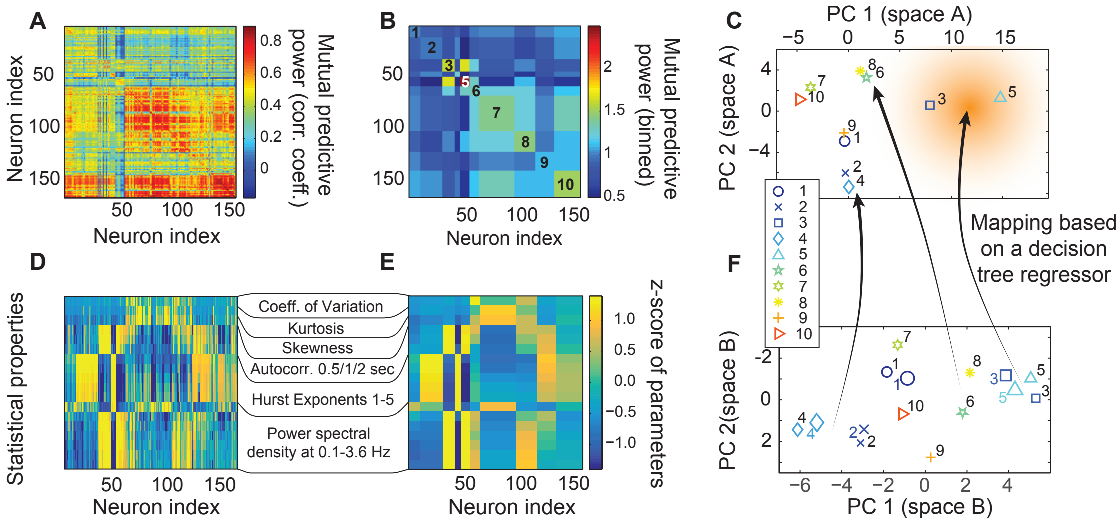

Figure 4: Using embedding spaces to choose datasets for focused retraining. **A.** Matrix of mutual predictive power, measured using the Pearson correlation coefficient between prediction and ground truth. **B.** Same as (A), but normalized for columns and binned to datasets. **C.** Principal component analysis applied to (B), keeping the first two PCs. **D.** Statistical parameters quantified for single neurons, standardized. **E.** Same as (D), but binned for datasets. **F.** 2D principal component space generated using (E). Symbols with numbers to the right are from training datasets, used to span the PCA space; symbols with numbers to the left are from the test dataset and were projected into the PCA space.

for the first layer, was chosen rather large, since simple CNNs with 3 or 4 convolutional layers with small input filter sizes (3, 5 or 7) performed poorly. No zero-padding was used. The numbers of filters were chosen to increase with depth in order to allow for a larger capacity to represent higher-order features. Standard *ReLU* activation units were used after each convolutional and dense layer, except for the last dense layer, where a linear activation was used to allow the output of continuous spiking probabilities.

All parameters were chosen based on intuition gained through a small exploratory hyperparameter study using diverse 3- and 4-layer CNNs with varying filter sizes, filter numbers and input window sizes. Overfitting was controlled by randomly omitting single neurons from the training data and checking predictive performance of the CNN model for the respective omitted neuron.

Although the above-described CNN performed well when it came to fitting single datasets of the ground truth, one single model trained on all datasets usually performed not as well for any of the datasets as the same CNN trained on the respective dataset alone. To better understand this, it was quantified how well a model that had been fitted to predict spikes for neuron i can make the same kind of predictions for neuron j. To this end, a low-capacity CNN (with two locally connected convolutional layers and one dense layer) was fitted for each neuron i. The small size of the network together with a high dropout rate during training (50% after each layer) was used to prevent overfitting. This model was then applied to predict spiking probabilities both for neuron i and all neurons $j \neq i$, resulting in a matrix of 'predictive power' (measured with the Pearson correlation coefficient between prediction and ground truth, identical to the evaluation of the spikefinder competition computed (Fig. S4A). For instance, row 55 shows how well spikes of neuron 55 can be predicted by the networks generated by all other neurons. Column 55, on the other hand, shows how well the model generated by neuron 55 can predict spikes of other neurons. The 5% neurons that were worst at predicting their own spiking were discarded from the following modeling, assuming bad recording quality that is not suited for inclusion into a training dataset.

Normalization over columns, symmetrization of the matrix and averaging over datasets yields a matrix of predictive power, i.e., a matrix of proximity in prediction-space between datasets (Fig. S4B). A PCA of this matrix results in an embedding space that was limited to two dimensions due to the low number of datasets. Datasets close to each other in the embedding space (e.g. 2 and 4) can predict each other's spikes very well, whereas datasets distant from each other in space (e.g. datasets 4 and 5) fail to do so. The idea behind this approach is very similar to the embedding spaces used by Team 8.

Using this approach, it is however not yet possible to map a neuron of a new dataset of

unknown properties onto the right location of the embedding space above. To solve this problem, the following statistical properties of the raw calcium time traces were calculated (Fig. S4D), in an approach that is similar to the long-range features of calcium traces used by Team 4:

- coefficient of variation, kurtosis, skewness
- autocorrelation of the calcium time trace with its future value in 0.5, 1 and 2 seconds
- generalized Hurst exponents of order 1-5
- the power spectral density at different frequencies between 0.1 and 3.6 Hz

We did not attempt to find a minimal set of predictive properties to reduce computation time here, but used dimensional reduction techniques to automatically extract the relevant independent components. After averaging the standardized values over datasets (Fig. S4E), we used the two first principal components to generate a map of proximity in statistical property space (Fig. S4F). This map was generated using the training datasets (numbers located on the right side of the symbols). Test datasets were mapped into this PCA space (numbers on the left side of the symbols).

To generate a mapping between the locations of the datasets in the two embedding spaces, a simple regressor (*DecisionTreeRegressor* from the scikit-learn package [13]) was fit to the training datasets (schematic arrows in Fig. S4C,F). We then used this mapping to determine the position of the test datasets in the embedding space of mutual predictive power.

Once the position in the embedding space is known for a dataset, the model that had been trained before on all datasets is retrained, but preferentially with neurons from datasets that lie close to the position in the embedding space. This preference was weighted with a function that decays exponentially over distance in the embedding space, as indicated by the red shading (Fig. S4C). Again, the functional form of the decay and the decay constant have been chosen heuristically without systematic optimization, since our goal was to showcase the power of our embedding space approach rather than finding a global optimum.

Embedding spaces as a visual and explicit intermediate step for model refinement are more easily accessible for users, allow the use of relatively small convolutional neuronal networks and can highlight similarities and differences between datasets. For example, it is interesting to see that in both embedding spaces, datasets 3 and 5 cluster together, whereas dataset 8, which uses the same calcium indicator (GCaMP6s) in the same brain region (V1), is in proximity of dataset 6 (GCaMP5k in V1). It was also observed that the datasets that use OGB-1 as indicator (1,2 and 4) tend to occupy similar regions of the embedding spaces.

This indicates that model selection is not only based on the calcium indicator and the brain region, but on hidden parameters, *e.g.*, signal-to-noise of the calcium recording, sampling rate, spike rate, temperature, indicator concentration, or others. To reliably comprise these possible hidden parameters with embedding spaces, it will be necessary to increase the number of datasets in order to support as many possible types of datasets as possible. However, the unknown dimensionality of this hidden parameter space makes it difficult to predict how many datasets would be required.

Code is available at
https://github.com/PTRRupprecht/Spikefinder-Elephant.

Team 6 — J. Friedrich, L. Paninski This algorithm approximates the calcium concentration dynamics $\boldsymbol{c}$ using a stable autoregressive process of order p (AR(p)).

$$c_t = \sum_{i=1}^{p} \gamma_i c_{t-i} + s_t. \tag{2}$$

The observed fluorescence $\boldsymbol{f} \in \mathbb{R}^T$ is related to the calcium concentration as [14]:

$$f_t = a\, c_t + b + \epsilon_t, \quad \epsilon_t \sim \mathcal{N}(0, \sigma^2) \tag{3}$$

where a is a non-negative scalar, b is a scalar offset parameter, and the noise is assumed to be i.i.d. zero mean Gaussian with variance σ^2. We assume units such that $a = 1$ without loss of generality.

The goal of calcium deconvolution is to extract an estimate $\hat{\boldsymbol{s}}$ of the neural activity $\boldsymbol{s}$ from the vector of observations $\boldsymbol{f}$. This leads to the following non-negative LASSO problem for estimating

the calcium concentration[15, 14]:

$$\underset{\hat{\boldsymbol{c}},\hat{\boldsymbol{s}}}{\text{minimize}} \quad \tfrac{1}{2}\|b\mathbf{1}+\hat{\boldsymbol{c}}-\boldsymbol{f}\|^2+\lambda\|\hat{\boldsymbol{s}}\|_1 \quad \text{subject to} \quad \hat{s}_t=\hat{c}_t-\sum_{i=1}^{p}\gamma_i\hat{c}_{t-i}\geq 0 \tag{4}$$

where the ℓ_1 penalty on $\hat{\boldsymbol{s}}$ enforces sparsity of the neural activity. Note that the spike signal $\hat{\boldsymbol{s}}$ is relaxed from non-negative integers to arbitrary non-negative values[14].

Problem (4) could be solved using generic convex program solvers, however, it is much faster to use OASIS [16], a dual active set method that generalizes the pool adjacent violators algorithm, a classic algorithm for isotonic regression [17]. The dual active set method yields an exact solution of Eq. (4) for $p=1$ and merely a greedy one for $p\geq 2$. Although an exact solution for the latter can be obtained by the primal active set method[15], here $p=2$ is used and the greedy but faster dual method which yielded similar scores (i.e. correlation values with ground truth).

The noise level σ is typically well estimated from the power spectral density (PSD) of $\boldsymbol{f}$ [18]. The parameters γ_i are either known a priori for a given calcium indicator or estimated from the autocovariance function of $\boldsymbol{f}$, and possibly improved by fitting them directly. The sparsity parameter λ can be chosen implicitly by inclusion of the residual sum of squares (RSS) as a hard constraint and not as a penalty term in the objective function [16, 18]. The dual problem

$$\underset{\hat{b},\hat{\boldsymbol{c}},\hat{\boldsymbol{s}}}{\text{minimize}} \quad \|\hat{\boldsymbol{s}}\|_1 \quad \text{subject to} \quad \hat{s}_t=\hat{c}_t-\sum_{i=1}^{p}\gamma_i\hat{c}_{t-i}\geq 0 \quad \text{and} \quad \|\hat{b}\mathbf{1}+\hat{\boldsymbol{c}}-\boldsymbol{f}\|^2\leq\hat{\sigma}^2T. \tag{5}$$

is solved by iterative warm-started runs of OASIS to solve Eq. (4) while adjusting λ, $\hat{b}$ (and optionally also γ_i) between runs until Eq. (5) holds. We refer the reader to [15] for the full algorithmic details.

The above parameter choices rely on a robust noise estimate $\hat{\sigma}$. The resampling of each spikefinder dataset to a fixed frame rate introduced artifacts into the data that corrupted the autocovariance and PSD such that it was not possible to obtain reliable noise and AR estimates based on the preprocessed data. Therefore, these parameters for baseline, sparsity and AR dynamics were determined based on the training data sets and kept fix for each test trace, thus not accounting for differences between neurons within one data set. Six parameters were fit: the percentile value and window length to estimate the baseline using a running percentile, the two AR coefficients, and the slope and offset of a linear function that determines the sparsity parameter λ as function of the noise. The latter was estimated on traces that were decimated by a factor of 10 to counteract the artifacts that had been introduced by upsampling the raw data.

Running OASIS with the known parameters directly yields an estimate $\hat{\boldsymbol{s}}$ of the neural activity. This estimate was already good for datasets 6-10, but noticeably improved for the first 5 datasets by convolving it with some kernel $\boldsymbol{k}$, to obtain the final estimate $\hat{\boldsymbol{s}}'=\hat{\boldsymbol{s}}*\boldsymbol{k}$. The kernel adjusts for mismatches between the actual calcium response kernel and the AR(2) model, smoothes the estimate, and accounts for the uncertainty of the exact spike timings by distributing spikes as spike rates over a few time bins. We used a kernel width of 30 bins and obtained it by averaging the closed form solutions of the least squares linear regression problem $\boldsymbol{k}=\arg\min_{\boldsymbol{k}}\|\hat{\boldsymbol{s}}*\boldsymbol{k}-\boldsymbol{s}\|^2$ for each true spike train $\boldsymbol{s}$ in the training set. Interestingly, the strategy used for hyperparameter optimization used here was very similar to that used by Team 1.

Because the evaluation criterion was correlation not the residual sum of squares, we considered to further optimize the kernel for this specific criterion using gradient decent initialized at the least squares solution; however, we did not obtain significant improvements.

Code is available at
https://github.com/j-friedrich/spikefinder_submission

Team 7 — M. Pachitariu, K. D. Harris This algorithm has been developed as part of Suite2p, a complete calcium processing pipeline [19]. This algorithm is called L0 deconvolution and consists of solving the following problem

$$\text{minimize } \|\boldsymbol{f}-\boldsymbol{s}*k\|^2+\lambda\|\boldsymbol{s}\|_0, \text{ such that } s_t\geq 0, \forall t, \tag{6}$$

where k is the calcium kernel (assumed, or estimated), $\boldsymbol{s}*k$ is the convolution of $\boldsymbol{s}$ and k, and $\|\boldsymbol{s}\|_0$ is the L_0 norm of $\boldsymbol{s}$, in other words the number of non-zero entries in $\boldsymbol{s}$. We do not describe

here the inference method for this model, but point the reader to our original derivation in [20]. This solution is approximate, due to its greedy nature. Exact solutions have been obtained by [21], in the case where the positivity constraint on s was removed, and the calcium kernel was restricted to be exponential.

The L_0 deconvolution model was developed as an alternative to the L_1-deconvolution model [14, 18, 15]. We originally believed that an L_0 penalty would better account for the binary nature of spike trains, and allow the algorithm to return sparse "spike trains". The algorithm indeed returns very sparse descriptions of the calcium data, which can deceptively look like electrophysiologically recorded spike trains. However, neither the L_0 nor the L_1 penalties are necessary or desirable for achieving best performance, the positivity constraint is sufficient [20].

Here, the training data was not used to set the parameters for the deconvolution (with the exception of time lags, see below). Instead, calcium kernels were chosen to be exponentials with timescales obtained from the literature for each specific sensor [22]. Following deconvolution, dataset-specific timelags were introduced for some of the spikefinder datasets. Also, the output was smoothed with a Gaussian kernel of a preset standard deviation (80ms for spikefinder data sets, 20 ms for GENIE datasets).

Code is available at `https://github.com/cortex-lab/Suite2P`.

Team 8 — B. Bolte The algorithm used for this submission consisted of a series of stacked convolutional neural networks with filter lengths of 10 to 100 milliseconds. The model was trained to maximize the Pearson correlation between the spike probabilities predicted by the model and the ground truth spike data. To capture the non-linear dynamic characteristic of this problem, additional features were added besides the raw calcium trace, including the first and second order derivatives, as well as quadratic features. Additionally, average pooling over convolutional filters was used to capture dynamics at multiple time scales.

During experimentation, it was observed that the spike behavior varied quite a bit in different data sets. From this observation, it was inferred that different convolutional filters would perform well for different data sets. To implement this idea, "data set embeddings" were used to weight the output of each convolutional filter during learning. A unique vector was learned for each data set, where the number of dimensions in the vector corresponded to the number of convolutional filters in the first layer of the model. The output of the first convolutional filter was weighted by it's corresponding vector.

Intuitively, these vectors represent embeddings for each data set, and the similarity between two embeddings represents the similarity of the spike behavior in each data set, since a model trained to infer spikes in the two data set would employ similar convolutional filters.

Code is available at `https://github.com/codekansas/spikefinder`.

Team 9 — T. Machado, L. Paninski The inference framework developed by Team 9 consists of two parts: a linear encoding model that takes in spikes and outputs simulated fluorescence traces (trained on paired spike train and fluorescence data), and a simple convolutional neural network to serve as a decoding model that outputs estimates of spikes given fluorescence observations and encoding model parameter estimates. This network is trained on large data sets simulated from the encoder model. The advantage of this approach is that we can train the decoder model to "saturation" by providing it as much training data as necessary to achieve good performance. On linear-Gaussian simulated data, the neural network decoder performed comparably to OASIS [15], a state-of-the-art inference method for efficiently efficiently solving the spike inference problem under linear-Gaussian assumptions (though both are fast enough to support online data analysis, OASIS runs significantly faster than the neural network decoder at test time).

Instead of directly using the *spikefinder* data sets to train a decoding algorithm, we generated simulated training data sets consisting of 5,000 traces, each of length 3,000 time steps. Each fluorescence trace was generated with the following second-order autoregressive model ($p = 2$):

$$c_t = \sum_{k=1}^{p} \gamma c_{t-k} + a s_t \tag{7}$$

$$f_t = c_t + b + \epsilon_t \tag{8}$$

The parameterization of each trace generated using Equation 8 was random. The noise was modeled as $\epsilon_t \sim N(0, \sigma_t^2)$. The jump size a of each spike was randomly sampled from a uniform distribution

between 0.5 and 1.5. Each simulated spike train, s, was sampled from a homogeneous Poisson process with a mean firing rate between 0 and 2 Hz. The baseline drift component, b, was modeled by low-pass filtering white noise. The baseline drift component significantly improved inference quality, in agreement with the observation of Team 1. In contrast, the statistics of the simulated spike trains, as well as the randomized jump sizes following each spike, had a much smaller impact on decoder performance.

The time constants of the autoregressive model, γ, and the scale of the noise, σ, were also sampled from random uniform distributions (such that a single decoder trained on these data could work well across multiple indicators and frame rates), but the precise parameterization was varied between model training sessions. However, in all cases, we found that a wide range of γ values could be learned by a single decoder model. For instance, we successfully trained decoder models across data with γ values spanning approximate decay times for fast OGB data recorded at 25 Hz, to slow GCaMP6S data recorded at 100 Hz. This shows that a single decoding model trained on simulated data can be used to analyze data produced by many different indicators and many different acquisition rates in agreement with Theis et al. [23]. Similarly, wide ranges of σ values could be learned by single decoders. However, there was a slight performance enhancement seen by training single encoders on mostly low SNR or high SNR data to improve performance on low SNR and high SNR real data, respectively.

Finally, in almost all cases, the second-order models (i.e. $p = 2$ in Equation 7) outperformed first-order models ($p = 1$). An exception were the OGB-1 data sets, as the sensor displays very rapid rise time kinetics following action potentials and has been previously shown to be especially well-described by first-order models[14].

To estimate the most likely spike train underlying a given fluorescence trace, we built a convolutional neural network. During training, the network was presented with f as well as parameter estimates for σ and γ given by methods published in earlier work [18]. Because the *spikefinder* data was upsampled from its native resolution and this introduced artifacts in the power spectrum of each fluorescence trace, we decimated each trace by a factor of 7-10 (depending on the approximate native time resolution of each dataset) before performing subsequent parameter fitting and analysis. The target of the network during training was the set of simulated spike trains, s, used to generate f using Equation 7.

A fairly simple architecture inspired by research into the construction of generative models for audio data was found to be effective [24]. In brief, the network consists of four 1D dilated convolutional layers containing 100 units, a filter size of 32, and rectified-linear (relu) nonlinearities. The first layer was dilated by a factor of 1, the second layer by 2, the third by 4 and the fourth by a factor of 8. Dropout (rate = 0.5) was also used at each layer. Finally, a fifth 1D convolutional layer with one unit, a filter size of one, and a relu nonlinearity was used to read-out a non-negative estimate of s from each f vector provided.

This architecture contained about 950,000 parameters and could be trained on a simulated data set of 5,000 traces in about 20 minutes (over 20 epochs) using the Google ML Engine. A single model trained on simulated data that spanned a wide range of σ and γ values performed well, but an ensemble of four models, each trained on a slightly different simulated data set, worked even better–as some decoders tended to work better or worse on each *spikefinder* data set. For our submission, we chose the decoding model that worked best for each dataset to use as our submission. For data set 5, which had high firing rates, we found that convolving the results with a small Gaussian kernel resulted in a modest improvement to our inference quality.

Code is available at
https://bitbucket.org/tamachado/encoder-decoder

Team 10 — D. Ringach This algorithm consists of a simple linear filter followed by a static-nonlinearity $f(t) = \phi(h(t) * s(t))$. The filter $h(t)$ is a linear combination of an even filter, estimating the mean of the signal at time t, and an odd filter, estimating the derivative of the signal at time t.

The even filter is a Gaussian, $h_{even}(\tau) = A\exp(-\tau^2/2\sigma^2)$, and the odd filter is the derivative of a Gaussian $h_{odd}(\tau) = Bt\exp(-\tau^2/2\sigma^2)$. The constants A and B are such that the norm of the filters is normalized to one, $\|A\| = \|B\| = 1$. These two filters are linearly combined while keeping the norm of resulting filter equal to one, $h(\tau) = \cos\alpha h_{even}(\tau) + \sin\alpha h_{odd}(\tau)$. The output nonlinearity is a rectifier to a power, $\phi(x) = (x - \theta)^\beta$ if $x > \theta$, and zero otherwise.

The model has only 4 parameters, $\sigma, \alpha, \theta, \beta$. The amount of smoothing of the signal is controlled

by σ, the shape of the filter is controlled by α, and the threshold θ and power β determine the shape of the nonlinearity. The model is fit by finding the optimal values of $\sigma, \alpha, \theta, \beta$ that maximize the correlation between its output $\hat{s}(t)$ and the recorded spiking of the neuron. Matlab's `fminsearch` was used to perform this optimization, which was typically finished in about 60 sec or less for most data sets. The only pre-processing done was a z-scoring of the raw signals. In one dataset (dataset 5, GCaMP6s in V1), an extra-delay parameter between the signal and the prediction was allowed.

Code is available at https://github.com/darioringach/Vanilla.

References

[1] Deneux T, Kaszas A, Szalay G, Katona G, Lakner T, Grinvald A, et al. Accurate spike estimation from noisy calcium signals for ultrafast three-dimensional imaging of large neuronal populations in vivo. Nature Communications. 2016;7:12190.

[2] Vogelstein JT, Watson BO, Packer AM, Yuste R, Jedynak B, Paninski L. Spike inference from calcium imaging using sequential Monte Carlo methods. Biophysical journal. 2009;97(2):636–55.

[3] Oord Avd, Kalchbrenner N, Kavukcuoglu K. Pixel recurrent neural networks. arXiv preprint arXiv:160106759. 2016;.

[4] Speiser A, Yan J, Archer E, Buesing L, Turaga SC, Macke JH. Fast amortized inference of neural activity from calcium imaging data with variational autoencoders. In: Advances in Neural Information Processing Systems. vol. 30; 2017. .

[5] Kingma DP, Welling M. Auto-encoding variational bayes. arXiv preprint arXiv:13126114. 2013;.

[6] Cho K, Van Merriënboer B, Bahdanau D, Bengio Y. On the properties of neural machine translation: Encoder-decoder approaches. arXiv preprint arXiv:14091259. 2014;.

[7] Glorot X, Bordes A, Bengio Y. Deep sparse rectifier neural networks. In: Proceedings of the Fourteenth International Conference on Artificial Intelligence and Statistics; 2011. p. 315–323.

[8] He K, Zhang X, Ren S, Sun J. Deep residual learning for image recognition. In: Proceedings of the IEEE conference on computer vision and pattern recognition; 2016. p. 770–778.

[9] Abadi M, Agarwal A, Barham P, Brevdo E, Chen Z, Citro C, et al. Tensorflow: Large-scale machine learning on heterogeneous distributed systems. arXiv preprint arXiv:160304467. 2016;.

[10] Graves A. Generating sequences with recurrent neural networks. arXiv preprint arXiv:13080850. 2013;.

[11] Handwriting Generation Demo in TensorFlow; 2015. Accessed: 2017-5-18. Available from: http://blog.otoro.net/2015/12/12/handwriting-generation-demo-in-tensorflow/.

[12] Chollet F, et al.. Keras. GitHub; 2015. Available from: https://github.com/fchollet/keras.

[13] Pedregosa F, Varoquaux G, Gramfort A, Michel V, Thirion B, Grisel O, et al. Scikit-learn: Machine Learning in Python. Journal of Machine Learning Research. 2011;12:2825–2830.

[14] Vogelstein JT, Packer AM, Machado Ta, Sippy T, Babadi B, Yuste R, et al. Fast nonnegative deconvolution for spike train inference from population calcium imaging. Journal of neurophysiology. 2010;104(6):3691–704.

[15] Friedrich J, Zhou P, Paninski L. Fast online deconvolution of calcium imaging data. PLoS Comput Biol. 2017;13(3):e1005423.

[16] Friedrich J, Paninski L. Fast active set methods for online spike inference from calcium imaging. In: Advances In Neural Information Processing Systems; 2016. p. 1984–1992.

[17] Barlow RE, Bartholomew DJ, Bremner J, Brunk HD. Statistical inference under order restrictions: The theory and application of isotonic regression. Wiley New York; 1972.

[18] Pnevmatikakis EA, Soudry D, Gao Y, Machado TA, Merel J, Pfau D, et al. Simultaneous Denoising, Deconvolution, and Demixing of Calcium Imaging Data. Neuron. 2016;89(2):285–299.

[19] Pachitariu M, Stringer C, Schröder S, Dipoppa M, Rossi LF, Carandini M, et al. Suite2p: beyond 10,000 neurons with standard two-photon microscopy. bioRxiv. 2016;.

[20] Pachitariu M, Stringer C, Harris KD. Robustness of spike deconvolution for calcium imaging of neural spiking. bioRxiv. 2017;p. 156786.

[21] Jewell S, Witten D. Exact Spike Train Inference Via L0 Optimization. arXiv preprint arXiv:170308644. 2017;.

[22] Chen TW, Wardill TJ, Sun Y, Pulver SR, Renninger SL, Baohan A, et al. Ultrasensitive fluorescent proteins for imaging neuronal activity. Nature. 2013;499(7458):295–300.

[23] Theis L, Berens P, Froudarakis E, Reimer J, Román Rosón M, Baden T, et al. Benchmarking Spike Rate Inference in Population Calcium Imaging. Neuron. 2016;90(3):471–482.

[24] Oord Avd, Dieleman S, Zen H, Simonyan K, Vinyals O, Graves A, et al. Wavenet: A generative model for raw audio. arXiv preprint arXiv:160903499. 2016;.
